# Seismic Signal Analysis Based on Variational Mode Decomposition and Hilbert Transform for Ground Intrusion Activity Classification

**DOI:** 10.3390/s23073674

**Published:** 2023-04-01

**Authors:** Yuan Sun, Dongdong Qian, Jing Zheng, Yuting Liu, Cen Liu

**Affiliations:** 1 College of Geoscience and Surveying Engineering, China University of Mining & Technology (Beijing), Haidian, Beijing 100083, China; 2 State Key Laboratory of Coal Resources and Safe Mining, China University of Mining & Technology (Beijing), Haidian, Beijing 100083, China

**Keywords:** ground intrusion activity, seismic signal analysis, variational mode decomposition, Hilbert transform, feature extraction, marginal spectrum, support vector machine

## Abstract

The identification of ground intrusion is a key and important technology in the national public security field. In this paper, a novel variational mode decomposition (VMD) and Hilbert transform (HT) is proposed for the classification of seismic signals generated by ground intrusion activities using a seismic sensing system. Firstly, the representative seismic data, including bicycles, vehicles, footsteps, excavations, and environmental noises, were collected through the designed experiment. Secondly, each original datum is decomposed through VMD and five Band-limited intrinsic mode functions (BIMF) are obtained, respectively, which will be used to generate a corresponding marginal spectrum that can reflect the actual frequency component of the signal accurately by HT. Then, three features related to the marginal spectrum, including marginal spectrum energy, marginal spectrum entropy, and marginal spectrum dominant frequency, are extracted for the analysis of the multi-classification using the support vector machine (SVM) classifier with the LIBSVM library. For the sake of testing and verifying the effectiveness of the proposed variational mode decomposition and Hilbert transform (VMD-HT) technique, the evaluation indicators including accuracy, precision, recall, and F1-Score are used and the results are compared with the time domain, frequency domain, ensemble empirical mode decomposition (EEMD), and empirical wavelet transform (EWT) combined with the HT analysis method. The performance of the VMD-HT method for ground intrusion activity classification provides an average value of 99.50%, 98.76%, 98.76%, and 98.75% for the four evaluation indicators, which are higher than all the other contrasted methods.

## 1. Introduction

Ground intrusion activities refer to illegal, unregistered, or prohibited users entering the designated area, thus causing acts endangering public security. For example, smuggling, drug trafficking, and illegal immigration in border areas. The theft of oil, gas, and chemical products through illegal vandalism and punching and theft in banks, vaults, museums, ancient tombs, and other places, and even some military actions that threaten national homeland security. Such intrusions pose a great threat to national security and the safety of people’s lives and property.

At present, the commonly used monitoring methods for intrusion mainly include infrared detection [1], pulse electronic fence [2], intelligent video analysis [3], Wi-Fi Networks [4], and microwave detection [5]. Although the above monitoring methods have achieved good intrusion detection results, there are still some limitations in terms of environment, electromagnetic interference, and concealment. The intrusion activities monitoring based on the seismic sensing system is to use the friction and impact between the intrusion activity and the ground to cause slight surface deformation, which will induce the seismic signal transmitted through the ground. The seismic sensing system can effectively respond to such stress and strain and convert the seismic signal into an electrical signal. In practice, the seismic sensor can be completely buried underground, closely coupled with the soil, and thus highly hidden from the surrounding environment. In addition, the seismic sensing system belongs to non-line-of-sight sensing and is not limited by topographic and geomorphic factors. Therefore, the seismic sensing system has the unique advantage of ground intrusion activity classification [6].

In recent years, many scholars have studied the classification and identification of intrusion signals using seismic sensing systems. It mainly includes the following cases: classification of different types of targets [7,8,9,10,11], such as human, animal, vehicle, excavation, etc. Classification of different vehicles [12,13,14], including light vehicles, heavy vehicles, wheeled vehicles, and tracked vehicles. Classification of human attributes [15,16,17], such as gender, height, weight, etc. There is also the classification of individual targets and noises [18,19,20]. The above-presented studies of various researchers reported that time-domain analysis cannot provide oscillation information on account of nonlinear and non-stationary intrusion signals, and the seismic waves propagate in different forms, directions, and speeds, leading to the complexity of seismic signal waveforms. Furthermore, frequency-domain analysis cannot observe the information about the frequency change of non-stationary seismic signals with time, and it provides the distribution of all signals in each frequency band due to the underlying non-stationary nature of the observed seismic signals. The seismic signals generated by ground intrusion activities have the characteristics of frequency varying with time. Therefore, the time-frequency analysis method can more deeply reflect the variation law of seismic signals. Many scholars have used time-frequency analysis methods to study the classification and recognition of ground seismic signals.

Short time-Fourier transform (STFT) [21,22,23] is one of the basic time-frequency analysis techniques and it has been used for the classification and identification of intrusion signals. Jinhui Lan et al. proposed a vehicle detection and noise removal solution based on STFT and solved the recognition problem for moving ground targets effectively [24], but STFT requires predefined window lengths, resulting in a fixed time and frequency resolution. In order to solve this problem, wavelet transform and wavelet packet transform that overcomes the fixed time-frequency resolution by adaptive windows are performed [25]. Wavelet transform has the problem of selecting the mother wavelet, which depends on the similarity between the mother wavelet and the signal to be analyzed. For this, Manish Kalra [26] proposed different modes of seismic signal extraction based on empirical wavelet transform (EWT), calculated statistical characteristics such as mean value, energy concentration, kurtosis, and entropy by EWT time-frequency coefficient, and obtained the classification accuracy of 83% for ground moving targets. For the sake of avoiding the selection of the parent wavelet function, Huang et al. proposed an empirical mode decomposition (EMD) method to construct an adaptive representation [27], and Kun Liu et al. decomposed the intrusion signal into intrinsic mode function (IMF) through EMD as a preprocessing step and then extracted the kurtosis feature. The proposed scheme can work well in extracting the feature vectors and in discriminating intrusion events [28]. Although EMD is a highly adaptive technique, it is an iterative algorithm without any theoretical and mathematical basis, and there is mode aliasing effect, which affects the performance and effect of classification. On the basis of EMD, ensemble empirical mode decomposition (EEMD) introduces white noise perturbation and ensemble average of signals, so as to avoid the problem of scale mixing and ensure the physical uniqueness of each component. Liu Zhiqiang et al. proposed an EEMD method combined with the advantages of principal component analysis to retain most of the relevant features [29]. Qu Hongquan et al. used the EEMD method to construct feature vectors with the energy ratio of each component of the IMF and then achieved ideal recognition accuracy [30].

Compared with EMD, the EEMD method and variational mode decomposition (VMD) method constructs the Wiener filter according to the center frequency of the component and takes the narrow-band property of the component into full consideration [31], so the frequency band of filtering is more concentrated, and the signal can be decomposed into components with coefficient characteristics adaptively [32]. With a solid theoretical foundation, the VMD method has been successfully applied in many fields such as seismic data analysis [33,34,35], Time-Varying system identification [36], structural health monitoring [37,38], Micro-Motion signal processing [39], fault detection and classification [40].

In this paper, a time-frequency analysis method based on VMD and Hilbert transform (HT) joint processing is proposed to extract the time-frequency-domain features of several typical ground intrusion seismic signals, including bicycles, vehicles, footsteps, excavations, and environmental noises, and then the classification of ground intrusion signal is studied by support vector machine (SVM) classifiers. The main contributions of this paper are summarized as follows:

- The self-developed continuous seismic data acquisition sensing system is introduced into the classification research of ground intrusion activities, which provides an intrusion monitoring method with concealability and non-invasion of personal privacy.
- A novel time-frequency technique combined with VMD and HT is proposed for seismic signals analysis. Three more representative features that can be further analyzed by the change rules of seismic signals are extracted, including marginal spectrum energy, marginal spectrum entropy, and marginal spectrum dominant frequency.
- The proposed variational mode decomposition and Hilbert transform (VMD-HT) method increases the classification accuracy, precision, recall, and F1-Score of ground intrusion signals compared with the time-domain analysis, the frequency-domain analysis, the ensemble empirical mode decomposition and Hilbert transform (EEMD-HT) time-frequency analysis, and the empirical wavelet transform and Hilbert transform (EWT-HT) time-frequency analysis method. We consider that the proposed method has broad application prospects for the classification of ground intrusion activity.

The rest of the paper is organized as follows: Section 2 describes the main theories, steps, and methods including the dataset, data preprocessing, VMD, HT, feature extraction, and classifier for the classification algorithm of ground intrusion activities. In Section 3, the comparison experiments of performance analysis with other features and methods are given, respectively. Finally, some conclusions and future work are presented in Section 4.

## 2. Methods

**Figure 1:**
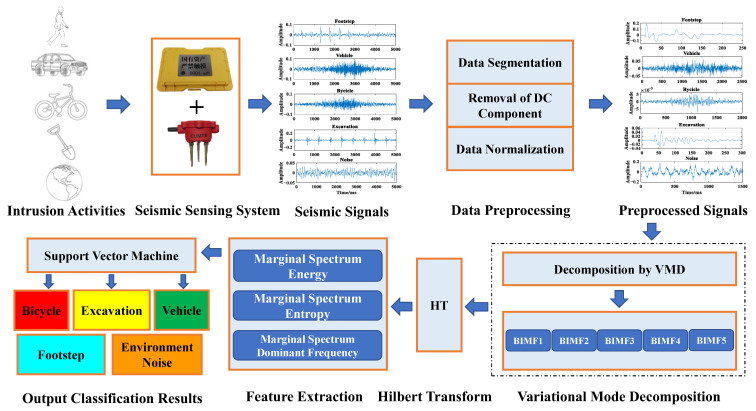
The algorithm flow chart of the classification for ground intrusion activities based on variational mode decomposition (VMD) and Hilbert transform (HT) time-frequency technique.

This section introduces the main steps involved in the classification algorithm of ground intrusion activities based on VMD and HT. As shown in Figure 1, it mainly includes experimental design and implementation of ground intrusion seismic signals acquisition, preprocessing of seismic signals, time-frequency-domain feature analysis and extraction based on VMD-HT, and the classification of different ground intrusion seismic signals by SVM classifier.

### 2.1. Dataset

**Figure 2:**
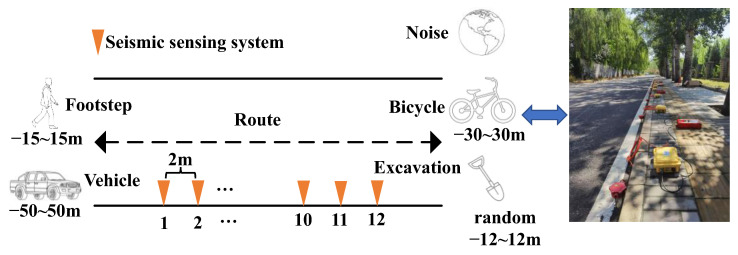
Experimental design and implementation of ground intrusion seismic signals acquisition using continuous seismic data acquisition sensing system.

The Continuous Data Acquisition System [41] is used for collecting the seismic signals generated by ground intrusion activities and the experimental design and implementation are shown in Figure 2. Twelve three-component moving-coil sensors with a natural frequency of 4.5 Hz and a sensitivity of 1.8 V/cm/s and collection stations with 1 KHz sampling frequency are arranged along the roadside in a straight line with 2 m spacing. Only one target moved near the sensor at a time during data acquisition and the trajectory of the controlled target is a straight line parallel to the road. To avoid the unacceptable differences in seismic energy between different target types, the effective data recording distance range of vehicles, bicycles, and personnel are (−50, 50 m), (−30, 30 m), (−15, 15 m), respectively. The excavation activities were carried out randomly within (−12, 12 m) along the route. The data set of ground intrusion seismic signals also includes the background noise of earth pulsation and the environmental noise in addition to the seismic signals of other targets during the experiment. The durations of seismic signals induced by bicycles, vehicles, footsteps, and excavations are different, the duration of vehicles is relatively long, and the footsteps and excavations are relatively short. Therefore, the segmentation lengths of different types of ground intrusion seismic signals are 2.5 s for vehicles, 2.0 s for bicycles, 0.25 s for footsteps, 0.3 s for excavations, and 1.5 s for noises, respectively. Finally, 1500 segmentations of data corresponding to each class are used for analysis.

### 2.2. Data Preprocessing

To improve the classification accuracy of ground intrusion seismic signals, it is necessary to preprocess the original signals, including data segmentation, removal of direct-current (DC) component, and data normalization. The data segmentation preprocessing operation is mentioned in Section 2.1.

The collected original seismic signal often contains a DC component due to the influence of the acquisition circuit. The frequency spectrum leakage of the DC component affects the accuracy of the low-frequency spectrum seriously. In order to ensure that the initial seismic energy of each acquisition unit is at the same level, it is necessary to remove the DC component of the original seismic signals before feature extraction and classification. The DC component is called the mean value in statistics. Therefore, this paper adopts the method of removing the mean value from the time domain to remove the DC component.

Since the energy of seismic signals generated by different intrusion activities are quite different, if the absolute value of energy is not converted into a relative relationship and the data with different energy sizes are directly input into the classifier, the classification accuracy will be affected. Here, we utilize the maximum-minimum normalization method to normalize the seismic data. After that, the value range of the original data is converted to an interval [0, 1]. As shown in Equation (1), X represents the original data, $X_{\max}$ and $X_{\min}$ represent the maximum and minimum values in the original data, and $X_{norm}$ is the data after normalization.

(1) $$X_{norm}=\frac{X-X_{\min}}{X_{\max}-X_{\min}}$$

### 2.3. Variational Mode Decomposition

The overall framework of VMD is variational problem, which is a completely non-recursive adaptive signal processing method. The band-limited intrinsic mode function (BIMF) is defined as an amplitude and frequency modulation signal, and its expression is described as follows:

(2) $$u_{k}(t)=A_{k}(t)\cos(\omega_{k}(t))$$

where $u_{k}(t)$ is the *k*-th BIMF, $A_{k}(t)$ is the instantaneous amplitude of $u_{k}(t)$, $\omega_{k}(t)$ is instantaneous frequency of $u_{k}(t)$.

The k modal functions $u_{k}(t)$ are sought through iterative search, and the sum of the estimated bandwidth of each mode is minimized on the premise that the sum of all BIMF components is the original signal. The following steps are utilized for the implementation of VMD:

(1) Perform Hilbert transformation to obtain the analytical signal of each modal function $u_{k}(t)$. Thus, the unilateral spectrum of each modal function is obtained, and the relevant analytic signal is as follows:

(3) $$S_{analytic}=(\delta (t)+\frac{j}{\pi t})*u_{k}(t)$$

where δ(t) is unit impulse function, j is an imaginary unit, t is the time-domain variable, and ∗ is convolution.

(2) Add an estimated center frequency $e^{-j\omega_{k}t}$ to the analytical signal of each mode and modulate the spectrum to the corresponding baseband:

(4) $$S_{base-band}=[(\delta (t)+\frac{j}{\pi t})*u_{k}(t)]e^{-j\omega_{k}t}$$

(3) Find the squared L2-norm of the gradient in Equation (4), then the variational problem can be expressed as follows:

(5) $$\underset{\left\{u_{k}\right\},\left\{\omega_{k}\right\}}{\min}\left\{\sum_{k}\partial_{t}{\left\|[(\delta (t)+\frac{j}{\pi t})*u_{k}(t)]e^{-j\omega_{k}t}\right\|}_{2}^{2}\right\}$$

(6) $$s.t.\sum_{k}u_{k}=f$$

where $\left\{u_{k}\right\}=\left\{u_{1},\ldots ,u_{K}\right\}$ and $\left\{\omega_{k}\right\}=\left\{\omega_{1},\ldots ,\omega_{K}\right\}$ are shorthand notations for the set of all modes and their center frequencies, respectively. Equally, $\sum_{k}=\sum_{k=1}^{K}$ is understood as the summation over all modes, $\partial_{t}$ represents the first partial derivative of a function with respect to time, and f is the decomposed signal.

The quadratic penalty factor α and Lagrange multiplication operator λ(t) are introduced to change the constrained variational problem into a non-constrained one. The expanded Lagrangian expression is as follows:

(7) $${\hat{u}}_{k}^{n+1}\left(\omega \right)=\frac{\hat{f}\left(\omega \right)-\sum_{i<k}{\hat{u}}_{i}^{n+1}\left(\omega \right)-\sum_{i>k}{\hat{u}}_{i}^{n}\left(\omega \right)+\frac{{\hat{\lambda }}^{n}\left(\omega \right)}{2}}{1+2\alpha {\left(\omega -\omega_{k}^{n}\right)}^{2}}$$

(8) $$\omega_{k}^{n+1}=\frac{\int_{0}^{\infty }\omega {\left|{\hat{u}}_{k}^{n+1}\left(\omega \right)\right|}^{2}d\omega }{\int_{0}^{\infty }{\left|{\hat{u}}_{k}^{n+1}\left(\omega \right)\right|}^{2}d\omega }$$

(9) $${\hat{\lambda }}^{n+1}(\omega )={\hat{\lambda }}^{n}(\omega )+\tau (\hat{f}(\omega )-\sum_{k}{\hat{u}}_{k}^{n+1}(w))$$

where ^ is the Fourier transform, ω is the frequency-domain variable, $i\in \left\{\left.K\right|i\neq k\right\}$, n is the number of iterations, and τ is the iteration step.

The VMD algorithm process is as follows:

(i) First, initialize $\left\{{\hat{u}}_{k}^{1}\right\}$, $\left\{\omega_{k}^{1}\right\}$, $\left\{{\hat{\lambda }}^{1}\right\}$, and n=0.
(ii) Execute Loop n=n+1.
(iii) Update ${\hat{u}}_{k}^{n+1}$, $\omega_{k}^{n+1}$, and ${\hat{\lambda }}^{n+1}$, according to Equations (7)–(9).
(iv) For the given discrimination accuracy e>0, if $\sum_{k}\frac{{\left\|{\hat{u}}_{k}^{n+1}-{\hat{u}}_{k}^{n}\right\|}_{2}^{2}}{{\left\|{\hat{u}}_{k}^{n}\right\|}_{2}^{2}}<e$, then stop iteration; otherwise, return to step (ii).

**Figure 3:**
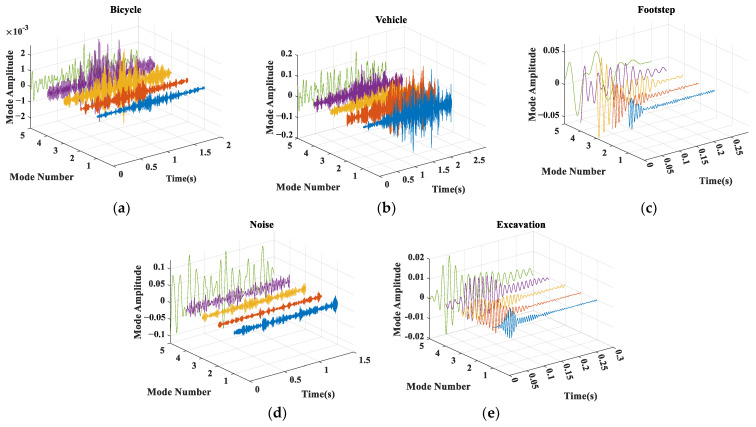
VMD of different ground intrusion seismic signals to obtain band-limited intrinsic mode function (BIMF). (**a**) Five BIMF components of one bicycle signal. (**b**) Five BIMF components of one vehicle signal. (**c**) Five BIMF components of one footstep signal. (**d**) Five BIMF components of one noise signal. (**e**) Five BIMF components of one excavation signal.

Before VMD of the preprocessed ground intrusion seismic signals, we first initialize the parameter α to 2000, the noise tolerance τ to 0, the maximum number of iterations to 500, and K to 5. Then, five BIMF components are obtained after VMD algorithm as shown in Figure 3.

### 2.4. Hilbert Transform

In order to analyze the change rule of intrusion seismic signals, Hilbert transform which has good time-frequency resolution and self-adaptability and can effectively avoid high-frequency interference in the analysis of nonlinear and non-stationary signal is carried out for each BIMF component after VMD decomposition. The marginal spectrum obtained by VMD-HT is used to reflect the actual frequency component information of the BIMF. The transformation process is as follows:

(10) $$\hat{u_{i}}(t)=H(u_{i}(t))=\frac{1}{\pi }\int_{-\infty }^{\infty }\frac{u_{i}(t^{\prime })}{t-t^{\prime }}d\tau =u_{i}(t)*\frac{1}{\pi t}$$

$u_{i}(t)(i=1,2,3,4,5)$ is the *i*-th BIMF component, $\hat{u_{i}}(t)$ is the Hilbert transform of $u_{i}(t)$, H is the Hilbert operator, 1/πt is the impulse response, t and $t^{\prime }$ is the time-domain variable, and ∗ is convolution. Then, the analytic function $z_{i}(t)$ of the original signal can be expressed as follows:

(11) $$z_{i}(t)=u_{i}(t)+j\hat{u_{i}}(t)=A_{i}(t)e^{j\theta_{i}(t)}$$

The instantaneous amplitude, phase, and frequency of analytic function, respectively, are as follows:

(12) $$A_{i}(t)=\sqrt{u_{i}^{2}(t)+{\hat{u_{i}}}^{2}(t)}$$

(13) $$\theta_{i}(t)=\arctan[\frac{\hat{u_{i}}(t)}{u_{i}(t)}]$$

(14) $$\begin{array}{l} \omega_{i}(t)=\frac{d\theta_{i}(t)}{dt} \end{array}$$

The Hilbert spectrum can be established as follows:

(15) H(ω,t)=Re[∑i=1KAi(t)ejωi(t)]=Re[∑i=1nAi(t)ej∫ωi(t)dt]

where Re[] means the real part of the signal. As can be seen from Equation (15), the signal can obtain a three-dimensional Hilbert spectrum of time-frequency-energy through HT. Frequency and amplitude are functions of time, and both are constants in Fourier analysis. So, HT is more suitable for analyzing the time-frequency characteristics of a nonlinear and non-stationary signal. By integrating H(ω,t) over time, the marginal spectrum can be further defined as follows:

(16) $$h(\omega )=\int_{-\infty }^{\infty }H(\omega ,t)dt$$

In a statistical sense, the marginal spectrum represents the cumulative amplitude distribution of each frequency point of the whole data. If there is a frequency of energy present in the signal, it means that there must be seismic waves of that frequency. In other words, marginal spectrum can reflect the actual frequency components more accurately. Compared with the existing signal feature extraction methods, it has better noise robustness [42]. In consideration of the mentioned advantages of marginal spectrum, this paper uses it as feature information to characterize the original seismic signals for ground intrusion activity classification purposes. The work related to feature extraction will be elaborated on in the next section.

### 2.5. Feature Extraction

Feature is a concise representation of signal. Feature extraction is to transform the target signal and extract feature information that reflects the essential attributes of the target. Its goal is to extract low-dimensional data that contains feature information from high-dimensional data [43]. For classification and recognition problems, feature extraction is an important step. The more representative, robust, and effective the extracted features are, the better the classification and recognition effect will be. According to the description in Section 2.3, marginal spectrum-related information is used to construct features. In the present article, three features, including marginal spectrum energy, marginal spectrum entropy, and marginal spectrum dominant frequency value, have been used to classify the different ground intrusion activities. The detailed introduction is as follows:

(1) Marginal Spectrum Energy [44]: The marginal spectrum energy is given by the following equation:

(17) $$E(\omega )=\int_{\omega_{1}}^{\omega_{2}}h^{2}(\omega )d\omega$$

$\omega_{1}$, $\omega_{2}$ is the starting and cutoff frequency of the marginal spectrum h(ω). The energy value is normalized as follows:

(18) $$NE_{k}=E_{k}/\sum_{k=1}^{K}E_{k}$$

NE is the normalized energy, and $E_{k}$ represents the energy value of the *k*-th BIMF component in Equation (18). The energy value is calculated by squaring the marginal spectrum which can reflect the actual frequency components. Therefore, the marginal spectrum energy enhances the actual frequency component of the seismic signals, which can suppress the influence of noise.

(2) Marginal Spectrum Entropy [45]: Shannon applied entropy to deal with information theory problems and defined information entropy as measure of uncertainty of the event, which is generally used to evaluate the complexity and irregularity of signal. The more complex and irregular the signal is, the higher the entropy value will be. As a kind of nonlinear index, marginal spectrum entropy has good ability to distinguish complexity and anti-interference. Hence, it is used to classify different ground intrusion activities that are defined in Equation (19):

(19) $$HHE=-\sum_{k=1}^{K}p_{k}\ln p_{k}$$

where $p_{k}=h(k)/\sum_{k=1}^{K}h(k)$ represents the probability of occurrence of the amplitude corresponding to the *k*-th frequency, and h(k) is the *k*-th marginal spectrum amplitude. The normalization of marginal spectrum entropy can be obtained:

(20) $$HE=\frac{HHE}{\ln L}$$

where L is the sequence length of h(k) in Equation (20).

**Figure 4:**
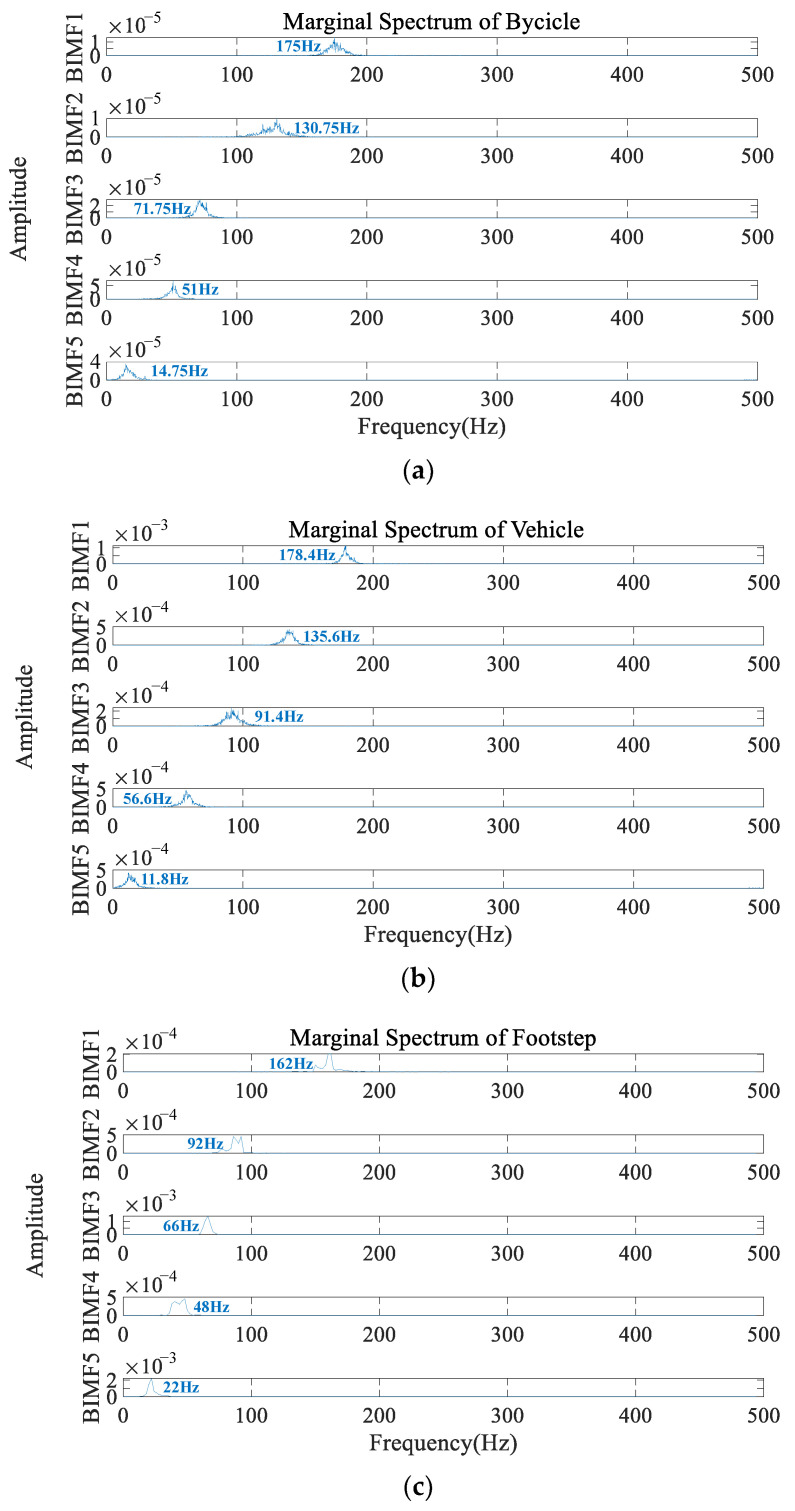

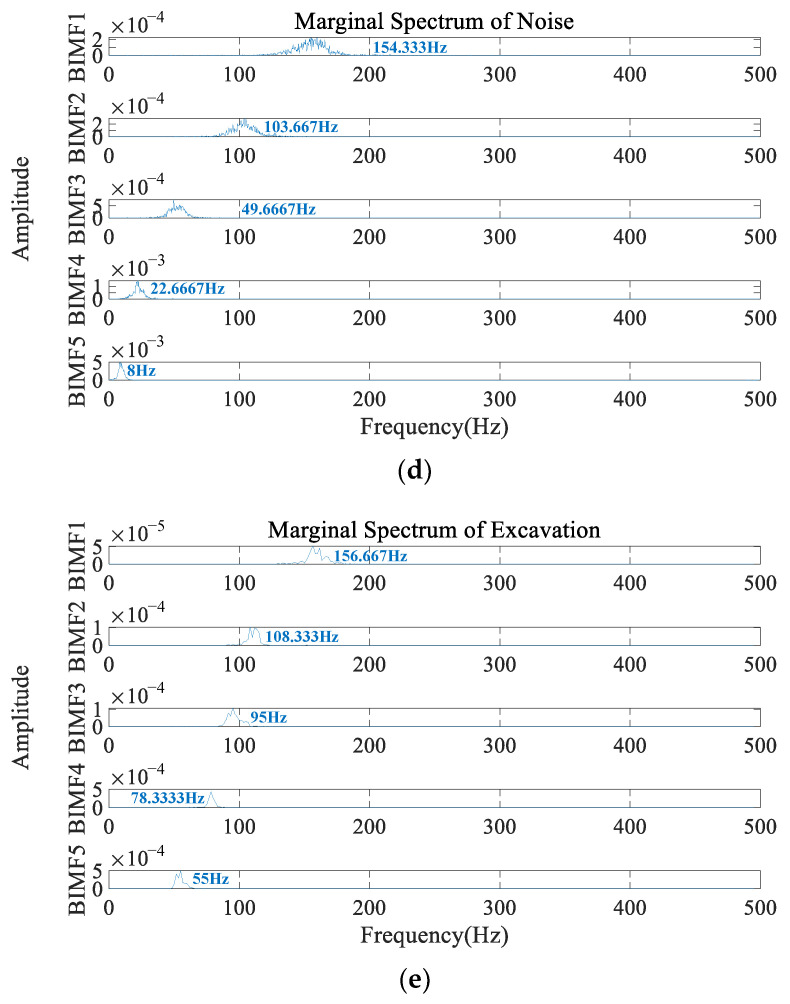
The marginal spectrum dominant frequency from each BIMF component from one of the seismic signals generated by different intrusion activities after variational mode decomposition and Hilbert transform (VMD-HT). (**a**) Bicycle. (**b**) Vehicle. (**c**) Footstep. (**d**) Noise. (**e**) Excavation.

(3) Marginal Spectrum Dominant Frequency [46]: The dominant frequency of marginal spectrum is the frequency corresponding to the maximum amplitude of the signal. The marginal spectrum dominant frequency of each BIMF component from one of the seismic signals generated by different intrusion activities after VMD-HT is shown in Figure 4. It is extracted and used for ground intrusion activity classification.

We obtain a total of 7500 samples dataset through the experiment in Section 2.1. To select the optimal SVM classifier hyperparameters and train and test the classifier model. We first divide the entire dataset into a training set and a testing set in terms of 70% and 30%. Therefore, the total number of training set samples is 5250 (5 classes, 1050 for each class), and the number of testing set samples is 2250 (5 classes, 450 for each class). Then, we introduce the method of five-fold cross-validation to divide the training samples into 5 copies, each of which serves as a cross-validation set (1050 samples in total, with a total of 5 classes, and 210 samples in each class), so as to obtain the optimal SVM classifier model. After that, the testing set samples will be used for evaluating the classification effect of the ground intrusion activities. Since the total number of BIMF is five for each sample, a total of 15(5×3) features for each sample are extracted in time-frequency domain. These features are then fed to the classifier for ground intrusion activities classification.

### 2.6. Classifier

The design of the classifier is an important part to distinguish ground intrusion activities accurately. A wide range of algorithms has been proposed for classification in the literature, which include, but are not limited to, maximum likelihood (ML), k-nearest neighbor (KNN), decision tree (DT), Gaussian mixture model (GMM), SVM, and artificial neural network (ANN). SVM [47,48] is the most commonly used model for seismic signal classification tasks [49].

The basic idea of SVM algorithm is to map samples in low-dimensional space to high-dimensional space through kernel function, construct optimal hyperplane, and find appropriate decision function to maximize the distance between samples separated by hyperplane. Introducing the parameter penalty factor c and relaxation variable $\xi_{i}$, the objective function and constraint conditions of the optimal hyperplane are as follows:

(21) $$\underset{w,b}{\min}\frac{1}{2}{\left\|w\right\|}^{2}+c\sum_{i=1}^{N}\xi_{i}$$

(22) $$y_{i}(w\cdot x_{i}+b)\geq 1-\xi_{i}$$

where w is the weight, b is the deviation, i=1,2,…,N is the number of training samples, $x_{i}$ is the *i*-th training sample, and $y_{i}$ is the classification result output.

The Lagrange function is as follows:

(23) $$L(w,b,\xi )=\frac{1}{2}{\left\|w\right\|}^{2}-\sum_{i=1}^{N}\alpha_{i}[y_{i}(w\cdot x_{i}+b)-1+\xi_{i}]$$

where $\alpha_{i}$ is the Lagrange multiplier corresponding to the *i*-th training sample. By converting the planning problem into the dual problem through the Lagrange function, the decision function of the hyperplane is obtained as follows:

(24) $$F(x)=\mathrm{sgn}\left(\sum_{i=1}^{N}y_{i}\alpha_{i}K(x_{i},x_{j})+b\right)$$

where $K(x_{i},x_{j})$ is the kernel function which represents the degree of similarity between the $x_{i}$ and $x_{j}$ samples. Since radial basis function (RBF) kernel function is not limited by the number of samples and feature dimensions, has fewer parameters to be determined, and less complexity, the SVM classifier in this paper adopts RBF kernel function, whose expression is as follows:

(25) $$K(x_{i},x_{j})=\exp{\left(-g\left\|x_{i}-x_{j}\right\|\right)}^{2}$$

g is the RBF kernel function parameter, which determines the sample interval scale range.

The classification of the ground intrusion activities in this paper is a multi-classification problem, so the LIBSVM [50] support vector library which establishes a classification hyperplane as a decision surface is introduced. The isolation edge between positive and negative examples can be maximized, which is a machine learning method developed in accordance with the principle of structural risk minimization. Based on the above methodology, the experimental results and analysis are presented in next section.

## 3. Experimental Results and Analysis

In this section, four evaluation indicators [51] are used to evaluate the excellent performance of the features extracted by the proposed VMD-HT time-frequency method and the other four methods including time-domain analysis, frequency-domain analysis, the EEMD-HT time-frequency analysis, and the EWT-HT time-frequency analysis. The four evaluation indicators are accuracy, precision, recall, and F1 scores, respectively. The expression of the four evaluation indicators is as follows:

(26) $$\mathrm{Accuracy}=\frac{TP+TN}{TP+FP+FN+TN}$$

(27) $$\mathrm{Precision}=\frac{TP}{TP+FP}$$

(28) $$\mathrm{Recall}=\frac{TP}{TP+FN}$$

(29) $$F1-\mathrm{score}=\frac{2*\mathrm{Recall}*\mathrm{Precision}}{\mathrm{Recall}+\mathrm{Precision}}$$

where *TP* means true positive, *TN* means true negative, *FP* means false positive, and *FN* is false negative. The accuracy is the percentage of correct results in the total sample. The precision represents the proportion of the number of correctly classified positive examples to the number of positive. The recall represents the proportion of the number of positive cases correctly classified to the actual number. F1-score is a comprehensive evaluation based on the harmonic average of recall and precision. In order to prove the effectiveness of the proposed VMD-HT time-frequency method, the results of a classification in a time domain and frequency domain and its features were compared with the time-frequency features extracted by the proposed method. At the same time, it was also compared with the time-frequency features extracted by EEMD, EWT time-frequency analysis method with HT.

### 3.1. SVM Model Parameter Selection

**Figure 5:**
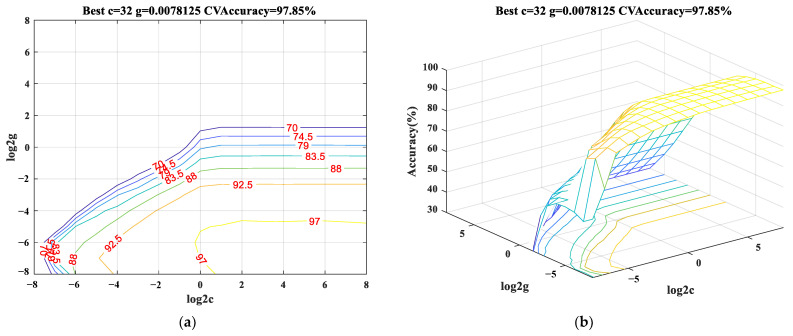
The support vector machine (SVM) model parameter selection for VMD-HT time-frequency-domain features training set (**a**) Contour map of SVM model parameter selection (**b**) 3D graphics of SVM model parameter selection.

In this paper, the radial basis kernel function with strong anti-interference to the noise was selected. Then, the grid search method was used to select the penalty parameter c and kernel function parameter g. As shown in Figure 5, we have used a quadratic SVM kernel with five-fold cross-validation to evaluate the performance of the algorithm. The penalty and kernel function parameters with the highest classification accuracy value in the VMD-HT time-frequency-domain features training set were used as the model parameters where c=32 and g=0.0078125. This method has also been used for other feature training set data.

### 3.2. Classification Performance of Time, Frequency, and Proposed VMD-HT Time-Frequency-Domain Features

The time domain includes 13 features such as mean value, kurtosis, and pulse factor. The frequency domain includes 13 features such as center-of-gravity frequency, root-mean-square frequency, and power spectrum density entropy. To analyze the superiority of the proposed VMD-HT time-frequency method, these features were input into the classifier model as feature vectors separately.

**Figure 6:**
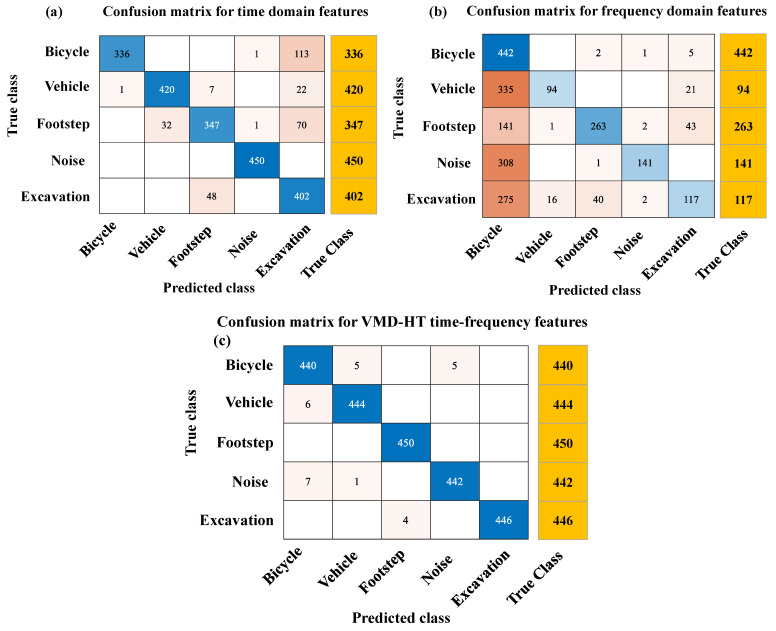
The confusion matrices obtained from feature testing sets generated in different domains. (**a**) Time-domain features. (**b**) Frequency-domain features. (**c**) VMD-HT time-frequency features.

**Figure 7:**
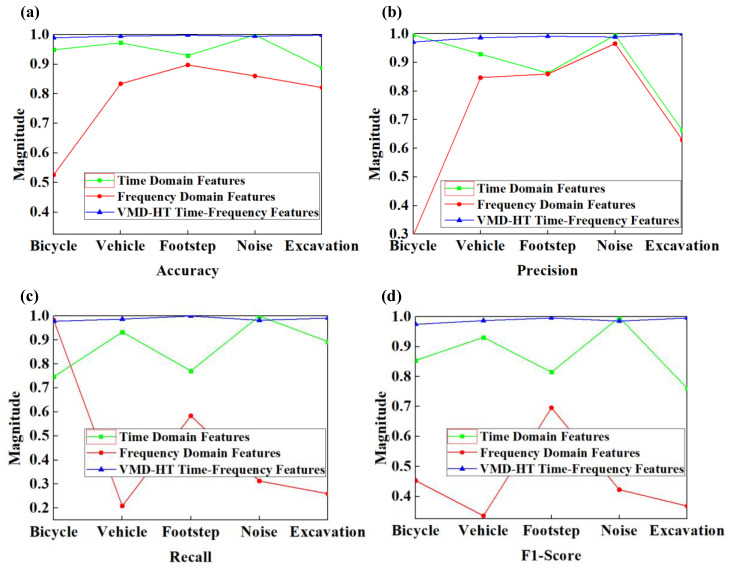
Evaluation indicators for classification effect of different domain features. (**a**) Accuracy for all classes. (**b**) Precision for all classes. (**c**) Recall for all classes. (**d**) F1-Score for all classes.

The confusion matrices are obtained from different domain features of the testing set, which were not included in the training set of the classifier model shown in Figure 6a–c. In the confusion matrix, the performance parameters including TP, TN, FP, and FN are presented and can be used to calculate the four evaluation indicators shown in Figure 7a–d. In comparison with other domain features, the VMD-HT time-frequency features produce a 25.25% improved accuracy in the classification of bicycles, 9.14% for vehicles, 8.44% for footsteps, 6.45% for noise, and 14.38% in the case of excavation. The improved precision, recall, and F1-Score for all classes are shown in Table 1.

Although it is slightly lower than the classification effect of time and frequency-domain features in some individual classes such as accuracy for noise and precision for bicycles, it can be seen from the comprehensive performance, as shown in Table 2. The average value of the four evaluation indicators increased by 4.74%, 9.81%, 11.87%, and 11.59% compared with time-domain features and increased by 20.71%, 26.85%, 51.80%, and 53.26% compared with frequency-domain features. The results fully illustrated that the proposed method produces an improved result for ground intrusion activities classification in comparison with others, especially in the frequency domain.

### 3.3. Classification Performance Compared with EEMD-HT and EWT-HT Methods

In order to demonstrate the excellent performance of the proposed VMD-HT time-frequency analysis method in the classification of ground intrusion activities. The EEMD and EWT methods were used for elaborated analysis in this section. According to the process of the VMD-HT algorithm, EEMD and EWT decomposition were performed on the preprocessed seismic data, and the IMF of EWT decomposition was set as five. Since the EEMD method cannot fix the number of decomposition components, we selected 5 IMF with the highest correlation to the original seismic signals after EEMD. Then, the HT transform was executed for the obtained IMF. Three features, including marginal spectrum energy, marginal spectrum entropy, and marginal spectrum dominant frequency, were extracted and the feature sets which would be input to the SVM classifier were constructed.

**Figure 8:**
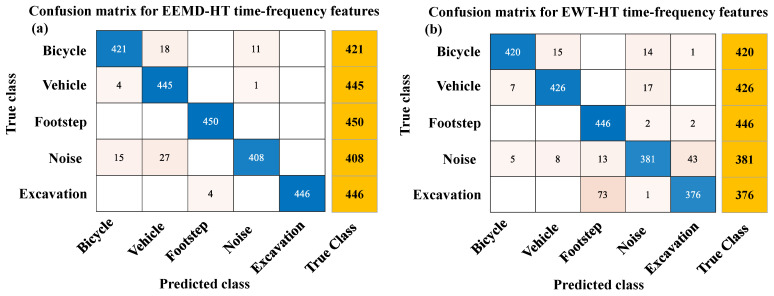
The confusion matrices obtained from testing sets generated by different time-frequency methods. (**a**) Ensemble empirical mode decomposition and Hilbert transform (EEMD-HT) time-frequency features. (**b**) Empirical wavelet transform and Hilbert transform (EWT-HT) time-frequency features.

**Figure 9:**
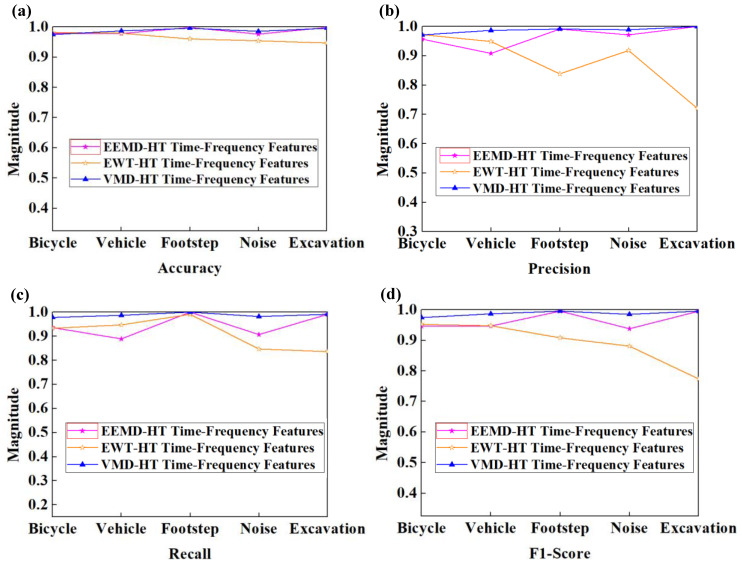
Evaluation indicators for classification effect of features generated by different time-frequency methods. (**a**) Accuracy for all classes. (**b**) Precision for all classes. (**c**) Recall for all classes. (**d**) F1-Score for all classes.

As shown in Figure 8, the confusion matrices of EEMD-HT and EWT-HT methods were obtained by using the feature testing set on the corresponding optimal SVM model, and the four evaluation indicators of the three time-frequency analysis methods were obtained through calculation displayed in Figure 9. As can be seen from Figure 9a, the classification accuracy by the three time-frequency analysis methods can all reach more than 96% in the five classes, and in the bicycle and footstep class, the classification accuracy of the EEMD-HT method is slightly higher than that of VMD-HT method proposed in this paper. As can be seen from Figure 9b, in terms of the classification accuracy index, the VMD-HT method is slightly higher than the EEMD-HT method in the other four classes except for vehicles. As shown in Figure 9c, the recall rates of the three methods are similar in the footstep class, and the performance of the VMD-HT method is more outstanding in the other four classes. Figure 9d also illustrates the excellent performance of the proposed method from the F1-score indicator. Although the EEMD-HT method and EWT-HT method also show good performance in some indicators and some classes, it can be seen from Table 3 that the VMD-HT method proposed in this paper has a certain improvement in average performance compared with the other two methods in terms of four evaluation indicators in all classes. As shown in Table 4, the VMD-HT time-frequency analysis method has shown the best performance in accuracy, precision, recall, and F1-Score for each class and the overall classification effect. The average value of the four evaluation indicators increased by 0.92%, 2.21%, 2.30%, and 2.30% compared with the EEMD-HT method and increased by 3.07%, 10.81%, 7.68%, and 9.48% compared with the EWT-HT method. It can also be seen that the features extracted by the time-frequency analysis method, including VMD, EEMD, and EWT can obtain better classification effects compared with the time or frequency-domain analysis method in the classification of ground intrusion activities.

## 4. Conclusions and Future Work

In this paper, we have proposed and investigated the potential of variational mode decomposition and Hilbert transform-based time-frequency analysis as an effective tool for ground intrusion activity classification using a continuous seismic data acquisition sensing system. VMD was used to decompose seismic signals of bicycles, vehicles, footsteps, excavations, and environment noises adaptively, and then the marginal spectrum that can more accurately reflect the actual frequency component was obtained through HT. Three features, including marginal spectrum energy, marginal spectrum entropy, and marginal spectrum dominant frequency, were extracted and input into the SVM classifier to classify ground intrusion activities. The experimental results showed that the VMD-HT method produced an average value of 99.50% for accuracy, 98.76% for precision, 98.76% for recall, and 98.75% for F1-Score, which are higher than time-domain analysis, frequency-domain analysis, EEMD-HT, and EWT-HT time-frequency analysis methods. The features extracted by the VMD-HT time-frequency analysis method are more representative than common time-domain and frequency-domain features, which can achieve better classification effects. Compared with the traditional time-frequency analysis methods of EEMD and EWT combined with HT for feature extraction and classification, the VMD-HT time-frequency analysis method shows the best performance and has broad application prospects in the classification of ground intrusion activities.

Based on this study, future work will focus on the classification of more than two seismic signals appearing simultaneously. In this paper, only one kind of intrusion activity occurs in the acquisition process. More than two kinds of intrusion activities will increase the complexity of seismic signals. How to separate different types of signals from mixed data and complete the classification will face challenges. However, this situation is more in line with the actual application and will be more meaningful for the classification and identification of intrusion activities. By arranging various observation systems, including cross array, diamond array, meter array, encircled array, etc., the positioning accuracy and error of different observation systems will also be studied.

## Figures and Tables

**Table 1:** The improvement of proposed VMD-HT method on each class and each evaluation indicator compared with other domain features.

	Bicycle	Vehicle	Footstep	Noise	Excavation
Accuracy	25.25%	9.14%	8.44%	6.45%	14.38%
Precision	32.56%	9.87%	12.99%	0.81%	35.26%
Recall	11.36%	41.59%	32.25%	32.55%	41.44%
F1-Score	32.11%	35.36%	24.06%	27.51%	43.13%

**Table 2:** Comprehensive classification performance of different domain features.

	Accuracy	Precision	Recall	F1-Score
Time-Domain Features	94.76%	88.95%	86.89%	87.16%
Frequency-Domain Features	78.79%	71.91%	46.96%	45.49%
VMD-HT Time-Frequency Features	99.50%	98.76%	98.76%	98.75%

**Table 3:** The improvement of proposed VMD-HT method on each class and each evaluation indicator compared with EEMD-HT and EWT-HT time-frequency methods.

	Bicycle	Vehicle	Footstep	Noise	Excavation
Accuracy	0.98%	1.63%	1.91%	2.91%	2.58%
Precision	0.68%	5.82%	7.65%	4.41%	6.03%
Recall	4.35%	1.90%	0.45%	10.50%	7.75%
F1-Score	2.54%	3.94%	4.37%	7.58%	11.09%

**Table 4:** Comprehensive classification performance of different time-frequency methods.

	Accuracy	Precision	Recall	F1-Score
EEMD-HT Time-Frequency method	98.58%	96.55%	96.46%	96.45%
EWT-HT Time-Frequency method	96.43%	87.95%	91.08%	89.27%
VMD-HT Time-Frequency method	99.50%	98.76%	98.76%	98.75%

## Data Availability

Data and materials are available on request.
